# Sol narae (Sona) is a *Drosophila* ADAMTS involved in Wg signaling

**DOI:** 10.1038/srep31863

**Published:** 2016-08-18

**Authors:** Go-Woon Kim, Jong-Hoon Won, Ok-Kyung Lee, Sang-Soo Lee, Jeong-Hoon Han, Orkhon Tsogtbaatar, Sujin Nam, Yeon Kim, Kyung-Ok Cho

**Affiliations:** 1 Department of Biological Sciences, Korea Advanced Institute of Science and Technology, 291 Daehak-ro, Yuseong-gu Daejeon, Korea

## Abstract

ADAMTS (a disintegrin and metalloproteases with thrombospondin motif) family consists of secreted proteases, and is shown to cleave extracellular matrix proteins. Their malfunctions result in cancers and disorders in connective tissues. We report here that a *Drosophila* ADAMTS named Sol narae (Sona) promotes Wnt/Wingless (Wg) signaling. *sona* loss-of-function mutants are lethal and rare escapers had malformed appendages, indicating that *sona* is essential for fly development and survival. *sona* exhibited positive genetic interaction with *wntless* (*wls*) that encodes a cargo protein for Wg. Loss of *sona* decreased the level of extracellular Wg, and also reduced the expression level of Wg effector proteins such as Senseless (Sens), Distalless (Dll) and Vestigial (Vg). Sona and Wg colocalized in Golgi and endosomal vesicles, and were in the same protein complex. Furthermore, co-expression of Wg and Sona generated ectopic wing margin bristles. This study suggests that Sona is involved in Wg signaling by regulating the level of extracellular Wg.

Proteases were originally started out as simple destructive enzymes in order to digest proteins and to provide amino acids to ancient organisms, but many proteases evolved in later times are specialized to change activity, localization, and binding properties of proteins and thereby affect many cellular functions. More than four hundred thousand proteases in all organisms can be classified into 9 categories and numerous subfamilies^1^,^2^. Among these proteases, ADAMTS family as a subclass of ADAM (a disintegrin and metalloproteases) family constitutes a group of zinc-dependent secreted proteases widely expanded during metazoan evolution, including 6 members in flies, 5 members in nematodes, and 19 members in mammals^3^.

These ADAMTSs are involved in many biological actions by processing mostly ECM and some non-ECM substrates. For example, ADAMTS-1 cleaves versican and aggrecan, and plays a key role in the ovulation process^4^,^5^. ADAMTS-2, 3, and 14 cleave procollagen I 6,7,8, and mutations in ADAMTS-2 cause Ehlers-Danlos syndrome, a connective tissue disorder^7^,^9^. ADAMTS-7 and 12 are significantly upregulated in arthritic patients^10^. Besides ECM proteins, ADAMTS-13 cleaves von Willebrand factor (vWF) in blood, and mutations in ADAMTS-13 result in thrombotic thrombocytopenic purpura (TTP)^11^. In addition, ADAMTSs either enhance or inhibit cancer development. The level of ADAMTS-7 is upregulated in carcinoma^12^ and ADAMTS-1 promotes tumor development through the induction of stromal reaction^13^. In contrast, ADAMTS-9 suppresses the formation of carcinoma by inhibiting angiogenesis^14^, and stable expression of ADAMTS-16 decreases proliferation of cancer cells^15^. Loss of ADAMTS-12 in mammals also increased tumor growth and progression^16^,^17^.

ADAMTS is synthesized as a zymogen and has a relatively long prodomain. The physical interaction between the prodomain and the metalloprotease domain is essential for the latency of enzyme activity^18^,^19^. Removal of prodomain in most ADAMTSs is mediated by furin, a proprotein convertase, in the secretory pathway^20^,^21^. However, prodomains of ADAMTS-9, -10, -15 are processed by furin in ECM^22^,^23^,^24^. In case of MIG-17 that is involved in male gonadal formation in *C. elegans*, the prodomain is cleaved autocatalytically^25^,^26^. Thus, the activation mechanism of ADAMTS family appears to be diverse and may be tightly controlled in order to ensure the generation of active forms at the right time and place.

We report here that an ADAMTS encoded by the *CG9850* gene in *Drosophila melanogaster* is capable of promoting Wnt/Wg signaling. Wnt family proteins are conserved morphogens for growth, development and adult homeostasis in all metazoans^27^,^28^. *CG9850* was named *sol narae* (*sona*) meaning ‘small wing’ in Korean, based on the small wing phenotype of mutant escapers. Fly Wg, a homolog of mammalian Wnt-1, is a prototype of Wnt family proteins essential for the development of all fly appendages, and the wing imaginal disc has been an excellent system to study Wg signaling. Wg is known to be secreted from Wg-producing cells at the dorsal-ventral (DV) midline in the wing pouch and forms a concentration gradient in extracellular matrix (ECM). Wg binding to Frizzled receptors on the plasma membrane of Wg-responding cells activates Wg signaling cascade, and Wg effector proteins including Sens, Dll and Vg are expressed in different regions of wing pouch^29^,^30^,^31^.

In this study, we focused on answering the following questions. Where and when is *sona* transcribed and translated? Where is the active form of Sona present? Which gene shows genetic interaction with *sona*? What are the *in vivo* roles of *sona*? We report here that *sona* exhibits a positive genetic interaction with *wntless* (*wls*) whose function is essential for secretion of Wg, and *sona* is positively involved in Wg signaling. Based on data provided in this report, we propose that Sona may modify proteins involved in Wg signaling.

## Results

### Sona is an essential ADAMTS

**Figure 1:**
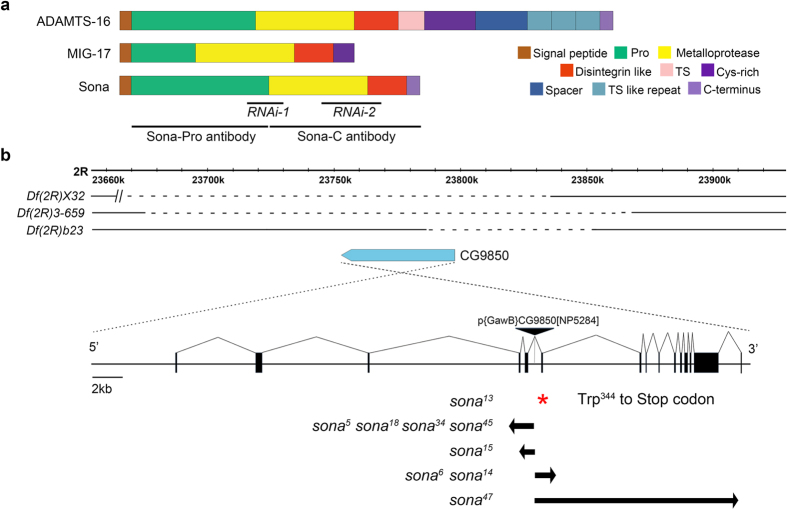
Domain structure of Sona and generation of *sona* mutants. (**a**) Domain structures of mammalian ADAMTS16, *C. elegans* MIG-17 and *Drosophila* Sona. The regions used to generate RNAi lines and those used to generate Sona-Pro antibody and Sona-C antibody were marked with black lines. (**b**) Three difficiency lines were shown with the genomic map of the region including the *sona* gene. The dashed lines represent the deleted regions in the difficiencies, and the *sona* gene is drawn as a pointed blue bar. Red asterisk indicates a mutation site of premature truncation in *sona*^*13*^ mutant, which was not produced by P element excision but by spontaneous mutation. The left and right arrows mark the region deleted in *sona* mutants by P element excision.

*Drosophila* has six ADAMTSs: Stall, Papilin, CG4096, CG6232, ADAMTS-A and CG9850 (Sona)^32^,^33^,^34^,^35^. Sona described in this study is Sona-PA, one of five Sona isoforms described in Flybase. Sona-PA protein shows ~45% similarity to mammalian ADAMTSs, and is composed of four domains: prodomain, protease domain, disintegrin-like domain and cysteine-rich domain. Members of ADAMTS family differ in the number of thrombospondin (TS) motifs, and some have unique carboxyl terminal domains. Sona is most similar to *Caenorhabditis elegans* MIG-17 involved in male gonadal formation^25^ (Fig. 1a; Supplementary Fig. S1), and *Bombyx mori* BmADAMTS-like that is induced in wing discs during pupal ecdysis^36^.

The *sona* gene is present in the 59F7-60A1 region at the tip of right arm in the second chromosome. P{GawB}CG9850[NP5284] flies used as a ‘*sona-Gal4*’ line in this study has a P element in an intron of the *sona* gene and was homozygous viable (Fig. 1b). However, homozygous larvae of P{GawB}CG9850 were occasionally lethal and much larger with extended larval stage at a low frequency (<1%). These larvae had tumorous imaginal discs as seen in *lethal giant larvae* (*lgl*) or *discs large* (*dlg*) mutants. To test if some P{GawB}CG9850 flies had *lgl* or *dlg* mutation, they were crossed with *dlg*^*m30*^, *dlg*^*m52*^, and *lgl*^*4*^ mutants for complementation test. Cross between P{GawB}CG9850 flies and *lgl*^*4*^ but not *dlg* yielded giant larvae, indicating that minor fraction of P{GawB}CG9850 flies had *lgl* mutation. To remove *lgl* and any other potential mutations, P{GawB}CG9850 was crossed with *w*^*1118*^ flies for four generations. The cleaned P{GawB}CG9850 line was homozygous viable without *lgl* phenotype. This line was crossed to *Δ2*–*3* for P element excision, and about 200 white-eyed flies were obtained. These white-eyed flies were crossed with deficiencies (*Df*(*2R*)*X32, Df*(*2R*)*23, Df*(*2R*)*3-659*) that uncover all or part of the genomic region containing the *sona* gene. Nine lines that failed complement these deficiencies were identified (Fig. 1b).

The regions deleted in the nine lines were identified by both RT-PCR and genomic PCR. All lines except *sona*^*13*^ had deletion in the exons of the *sona* gene, and *sona*^*13*^ had a point mutation that changed Trp344 to stop codon and incomplete excision of the P element. On the contrary, ten other white-eyed lines that were complemented by the deficiency lines did not have deletion in the *sona* coding region. We further cleaned up *sona* deletion mutants by crossing them with *w*^*1118*^ for four generations in order to remove any unwanted mutations that might have been generated during the process of imprecise excision of the P element.

### Sona is essential for survival and development of flies

**Figure 2:**
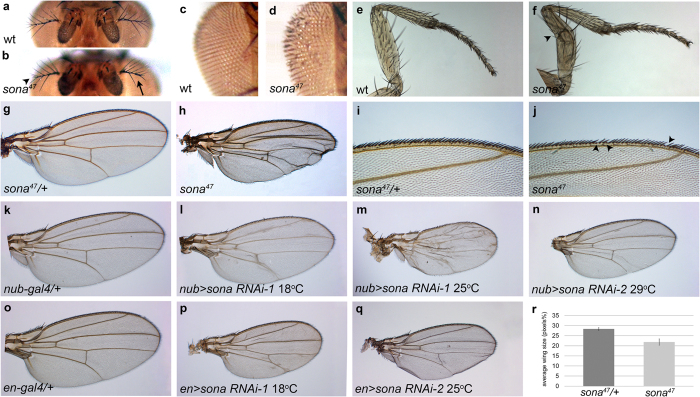
Sona is essential for the development of fly appendages. These phenotypes were observed from escapers of *sona*^*47*^, *sona*^*13*^, and *sona*^*18*^ as well as transheterozygotes. (**a**,**b**) Arista in *w*^*1118*^ as a wild-type (wt) control (**a**), and missing and malformed branches of arista in *sona*^*47*^ escapers are marked with arrow and arrowhead, respectively (**b**). (**c**,**d**) Disrupted ommatidial bristles in *sona* (**d**) compared to control (**c**). (**e**,**f**) Kinked femur of *sona* (arrowhead in **f**) compared to control (**e**). (**g,h**) Wings of *sona*^*47*^ (**h**) are smaller than those of *sona* heterozygotes (**g**). (**i,j**) Missing bristles at the anterior wing margin of sona^47^ (**j**) compared to the heterozygous wing (**i**). (**k–n**) Wing size becomes smaller by expression of *sona RNAi-1* (**l,m**) and *sonaRNAi-2* (**n**) by *nub-Gal4*. Wings become crumpled in severe cases (**m**). (**o–q**) Wings become smaller by expression of *sona RNAi-1* (**p**) and *sonaRNAi-2* (**q**) by *en-Gal4*. (**r**) Quantitative analysis of the wing size (n = 10 each) shown in (**g**,**h)**. Genotypes are indicated at lower left.

All *sona* mutants exhibited lethality throughout larval and pupal stages. Three *sona* mutants, *sona*^*13*^, *sona*^*18*^, and *sona*^*47*^, were chosen for further analysis. Rare escapers from these *sona* mutants could reach adulthood at a very low frequency (<1%), and exhibited various developmental defects. The percentage of escapers and their morphological defects were overall similar in the *sona* mutants. Branches in their arista were partially missing or malformed (Fig. 2a,b), and the interommatidial bristles were disoriented in dorsal eye (n = 31; Fig. 2c,d; Supplementary Fig. S2). 40% of the legs had kinks in the femur (n = 36; Fig. 2e,f), and loss of tarsal claw was occasionally observed (not shown). Their wings were crumpled and ~25% smaller than *sona* heterozygous wings (n = 26; Fig. 2g,h,r). Their wing margin bristles were missing in 80% of wings with average 2.7 anterior bristles (n = 26) (Fig. 2i,j).

We also generated two *UAS-sona RNAi* lines by using two different regions of the *sona* cDNA (Fig. 1a; Supplementary Fig. S1). Expression of these two lines by various *Gal4* lines induced similar phenotypes such as lethality and malformation of appendages (Supplementary Table S1). Thus, the *sona RNAi* phenotypes were due to the loss of *sona* function but not off-target effects. For instance, expression of *sona RNAi-1*^*11-4*^ (*sona RNAi-1* hereafter) driven by *nubbin* (*nub*)*-Gal4* at 18 °C and 25 °C resulted in small and crumpled wings, and similar wing phenotype was induced by expression of *sona RNAi-2*^*23-2*^ (*sona RNAi-2* hereafter) at 29 °C (Fig. 2k–n). When *sona RNAi-1* and *sona RNAi-2* were driven by *engrailed* (*en*)*-Gal4* at 18 °C and 25 °C, respectively, rarely obtained adults had smaller wings (Fig. 2o–q). In summary, *sona RNAi-1* induced stronger phenotype than *sona RNAi-2*, and *Gal4* lines that drive expression of *sona RNAi* during embryonic stages induced lethality while those during later stages of development induced loss or malformation of appendages (Supplementary Table S1).

### The lethality of *sona* mutants can be rescued by overexpression of Sona

Similar to the phenotypes induced by *sona RNAi* expression, overexpression of Sona resulted in lethality or defects in appendages (Supplementary Table S1). This indicated that protease activity of Sona must be tightly regulated for proper development and survival. To test whether the protease activity of Sona is essential for its role *in vivo*, we generated *UAS-sonaE475A*, which encodes the protease-dead form of Sona by substituting glutamic acid to alanine at 475^th^ residue in the conserved site for protease activity (Supplementary Fig. S1). Expression of SonaE475A resulted in neither lethality nor any developmental defects, demonstrating that the protease activity of Sona is essential for the role of Sona *in vivo* (Supplementary Fig. S3 and Table S1) unlike five mammalian ADAMTS-like (ADAMTSL) proteins that have no protease activity^3^,^37^.

To prove that the deletion in the *CG9850* was responsible for phenotype of *sona* mutants, we checked if the lethality of *sona*^*47*^ can be rescued by expressing *UAS-sona* with various *Gal4* drivers (Supplementary Table S2). When *UAS-sona sona*^*47*^/*CyO* was crossed with *nub-Gal4 sona*^*47*^/*CyO-GFP* and cultured at 18 °C, about 36% of *UAS-sona sona*^*47*^/*nub-Gal4 sona*^*47*^ were obtained as normal adults. When *UAS-sona sona*^*47*^/*CyO-GFP* were crossed with *ptc-Gal4 sona*^*47*^/*CyO-GFP* and cultured at 18 °C, 23% of *UAS-sona sona*^*47*^/*ptc-Gal4 sona*^*47*^ adults were obtained as normal adults. Two important conclusions were obtained from this rescue experiment. First, *CG9850* is the *sona* gene. Second, the Sona-PA form is sufficient to rescue the lethal phenotype of *sona* mutants.

### Transcription and translation of Sona are dynamically regulated during development

**Figure 3:**
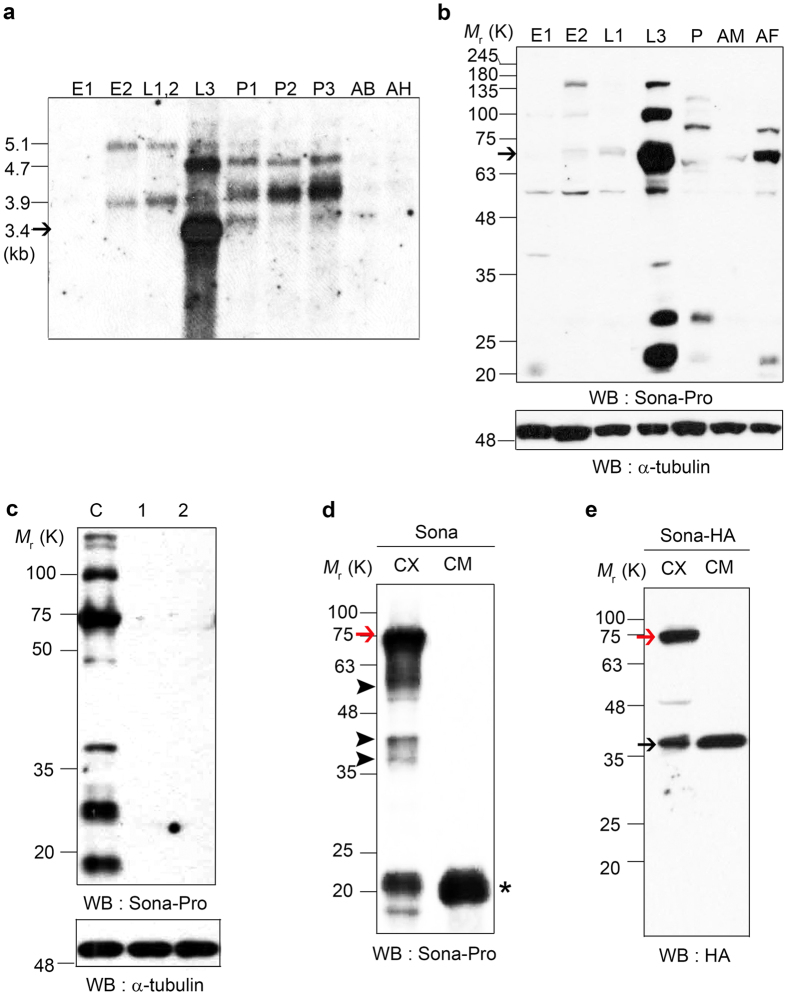
Transcriptional and translational expressions of Sona during development and active form of Sona in S2 cell culture. (**a**) Expression of *sona* transcripts during development detected with the probe representing 732^th^ to 1920^th^ nucleotides of the *sona-RA* cDNA. *sona-RA* transcript is marked with an arrow. E1, AEL (After egg laying) 0~12 hr; E2, AEL 12~24 hr; L1,2, first and second instar larvae; L3, late third instar larvae; P1, early pupae; P2, mid pupae; P3, late pupae; AB, adult body; AH, adult head. (**b**) Expression of Sona protein during development. E1, AEL 0.5~11 hr; E2, AEL 16~24 hr; L1, first instar larvae; L3, late third instar larvae; P, late pupae; AM, adult male; AF, adult female. The arrow indicates the full-length Sona-PA form. (**c**) The cellular extract was obtained from the late 3^rd^ instar larvae raised at 18 °C and then at 29 °C for 24 hrs until dissection. **C** is *actin-Gal4* (*act*) >*Gal80*^*ts*^, (**1**) is *act* > *sona RNAi-1; Gal80*^*ts*^, and **2** is *act* > *sona RNAi-2; Gal80*^*ts*^. (**d**,**e**) Sona proteins in the cellular extract (CX) or conditioned media (CM) of *sona* (**d**) or *sona-HA* (**e**) S2 cells. Red arrows and black arrows mark full-length Sona and activated Sona, respectively. Asterisk marks the 22 kDa prodomain fragments. Arrowheads indicate intracellular fragments of Sona.

As a first step toward understanding the role of Sona during development, we carried out both Northern and Western analyses to examine the expression pattern of *sona* mRNA and protein. Multiple *sona* transcripts were expressed during development, and a 3.4 kb transcript in the late 3^rd^ instar larval stage was most abundant (Fig. 3a). The amino acid sequence of Sona-PA reported in Flybase was identical to those deduced from a 2.97 kb *sona* cDNA that had been isolated from a late 3^rd^ instar larval cDNA library, so the 3.4 kb transcript may encode the Sona-PA form. We focused on the Sona-PA form in this study.

We generated two antisera, ‘Sona-Pro’ with the prodomain of Sona as an antigen in rabbit and ‘Sona-C’ with the carboxyl region of Sona as an antigen in mouse as marked in Fig. 1a, and purified them by affinity purification. All analyses were carried out with the purified antibodies. Western analysis with Sona-Pro antibody showed that multiple forms of Sona are dynamically expressed during development (Fig. 3b). For instance, the level of Sona was highly increased in the late third instar stage, which was corresponding to the increased level of *sona* transcripts in the same stage. 70 kDa Sona protein was most abundant in the late 3^rd^ instar larval stage (arrow in Fig. 3b), imlying that 70 kDa band is most likely the Sona-PA form (Fig. 3a).

To test whether multiple bands in the Western blot were authentic Sona proteins, *UAS-sona RNAi-1* and *UAS-sona RNAi-2* were crossed with *actin-Gal4*, cultured at 18 °C to the third instar stage, shifted to 29 °C for 24 hours, and the larval extracts were prepared. No protein bands were identified with Sona-Pro antibody in the larval extracts. Thus, the bands in the control extract are either different isoforms or processed forms that contain all or some portion of the prodomain (Fig. 3c). Furthermore, full-length Sona was absent in homozygous *sona*^*13*^, *sona*^*18*^ and *sona*^*47*^ larvae (Supplementary Fig. S4). The *sona*^*47*^ extract had a smaller fragment that may be the truncated Sona protein. Sona-C antibody was not suitable for Western analysis.

### Active Sona form is present in both intra- and extra-cellular regions

Because ADAMTSs are secreted proteases, we examined if active form of Sona is secreted. An *S2* cell line that constitutively expresses *sona* cDNA was generated (Materials and Methods) and used to obtain cellular extract (CX) and conditioned media (CM) for Western analysis. Sona-Pro antibody detected both full-length 70 kDa Sona and smaller fragments in CX but only 22 kDa prodomain-containing fragments in CM, indicating that the full-length Sona is not secreted (Fig. 3d). To detect the active form of Sona, we constructed a *sona* cDNA tagged with HA in front of the stop codon and established *S2 sona-HA* cell line. Both CX and CM from the culture of this cell line were analyzed with anti-HA antibody (Fig. 3e). The 37 kDa form was detected in both fractions as an active form devoid of the prodomain. This demonstrated that active Sona is present in both intracellular and extracellular regions.

### Sona is expressed in discrete regions and can diffuse far from the expressed site in imaginal discs

**Figure 4:**
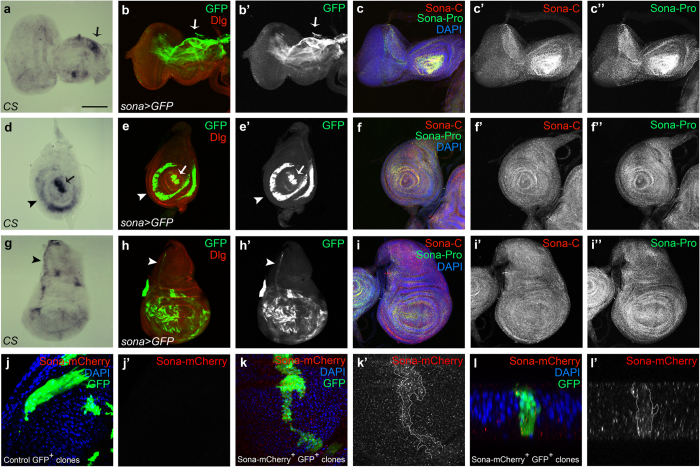
Expressions of *sona* transcripts and Sona proteins in flies and S2 cells. Eye-antenna discs (**a**–**c**), leg discs (**d**–**f**) and wing discs (**g**–**i**) from late third instar larvae were shown. (**a**,**d**,**g**) *in situ* hybridization with *sona* cDNA as a probe. Arrows and arrowheads indicate the region with high level of *sona* transcripts. (**b**,**e**,**h**) The pattern of GFP expressed by *sona-Gal4*. Discs-large (Dlg) was used as membrane marker. (**c**,**f**,**i**) The localization pattern of Sona visualized with two Sona antibodies, Pro- and C-antibodies. (**j–l**) Patterns of Sona-mCherry produced from a Sona-mCherry^+^ clone in a wing disc (**k**) or that of control (**j**). The Sona-mCherry^+^ clone is outlined in (**k’**). A cross section view of the same clone in (**k**) at the DV boundary (**l**). Black and white images of GFP, Sona-C, Sona-Pro or Sona-mCherry are shown. Scale bars: (**a–i**) 100 μm; (**j**,**k**) 30 μm; (**l**) 9.5 μm.

We checked expression pattern of *sona* transcripts in imaginal discs by *in situ* hybridization and the pattern of GFP driven by *sona-Gal4*. In the eye-antenna disc of *sona* > *GFP*, GFP was expressed at a high level in dorsal peripodial epithelium (arrow in Fig. 4b) and in photoreceptor clusters (Supplementary Fig. S5a–d). In the leg disc, GFP was expressed in the presumptive region of claw, tibia, and femur^38^ (arrow and arrowheads in Fig. 4e). In the pouch of wing discs, *sona* was expressed in a complicated mosaic pattern (Fig. 4h). Although the expression pattern of *sona* transcripts was not at high resolution, it was in accordance with the pattern of *sona* > *GFP* (arrows and arrowheads in Fig. 4a,d,g).

We then examined the expression pattern of Sona protein in imaginal discs with both Sona-Pro and Sona-C antibodies that had been tested for their specificity (Supplementary Figs S5 and S6). Sona was more or less evenly distributed except some regions with a higher level of Sona (Fig. 4c,f,i). At higher magnification, Sona was highly enriched in the apical region of photoreceptor clusters (Supplementary Fig. S5a–d) and in the disc proper of wing discs, and was present at a negligible level in *sona* mutants (Supplementary Fig. S5e–h).

Because Sona protein was ubiquitously present in entire discs in contrast to the localized pattern of *sona* transcripts, we thought that Sona may be efficiently diffused from the expressed site. To check how far Sona can diffuse, we generated *UAS-sona-HA* and *UAS-sona-HA-mCherry* (hereafter ‘*UAS-sona-mCherry*’) flies using *sona* cDNA tagged with HA or HA-mCherry in front of the stop codon. These *UAS* lines driven by various *Gal4* lines could induce lethality and structural defects, indicating that the tags did not compromise the protease activity of Sona (Supplementary Fig. S7). We generated clones expressing Sona-mCherry by the flp-out method^39^ and detected both intra- and extra-Sona-mCherry proteins far from the GFP^+^ Sona-mCherry^+^ clone (Fig. 4k). The cross-section view of the same clone also showed the diffusion of Sona-mCherry proteins away from the clone (Fig. 4l). mCherry signals were genuine because no signal was detected in and around a control GFP^+^ clone at the same level of laser intensity (Fig. 4j). We proved that the mCherry signal is not from the mCherry tag cleaved off from Sona by Western analysis, in which Anti-HA antibody recognized full-length and active form of Sona-HA and Sona-HA-mCherry in CX and CM, respectively, but not any other smaller bands (Supplementary Fig. S8). Thus, our data demonstrated that Sona can diffuse far from the source. How Sona can diffuse and which form of Sona can diffuse need further studies.

### Carboxyl region and prodomain of Sona are not colocalized in ECM

**Figure 5:**
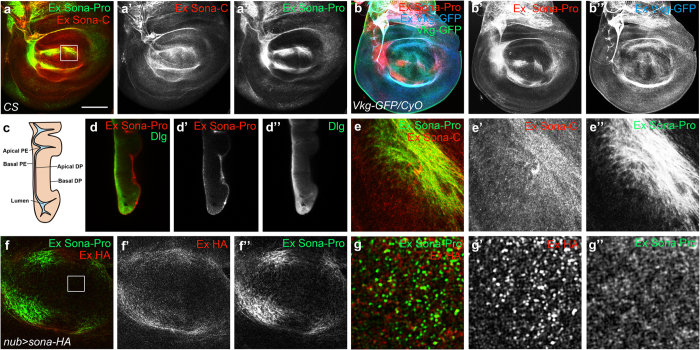
Active Sona is present in ECM. (**a**) Extracellular patterns of Sona in the basal ECM of disc proper. Sona-Pro and -C antibodies detected overlapping structures. The region marked with a square was magnified in (**e**). (**b**) Extracellular patterns detected by Sona-Pro antibody in Collagen IV GFP-trap line, *viking*^*G454*^(*Vkg-GFP*/*CyO*). GFP signal (green) was detected as representing both intracellular and extracellular Viking-GFP. To detect only the extracellular Viking-GFP, rabbit anti-GFP antibody and Cy5-conjugated secondary antibody were used with extracellular staining method in (**b”**). (**c**) A diagram of peripodial epithelium (PE) and disc proper (DP) layer of a wing disc. (**d**) A cross-section image of a wing disc with the extracellular pattern shown with Sona-Pro antibody. Intracellular Dlg that is enriched at the septate junction was counterstained to show the structure of the wing disc. (**e**) The magnified image of the marked region in (**a**), showing the difference in the pattern recognized by Sona-Pro and Sona-C antibodies. (**f**,**g**) Extracellular patterns of HA-tagged Sona and Sona prodomain visualized with HA and Sona-Pro antibody in a *nub* > *sona-HA* disc. The region marked with a square in (**f**) was magnified in (**g**). Scale bars: (**a**,**b**,**d**) 100 μm; (**e**) 18.8 μm; (**f**) 40 μm; (**g**) 6.5 μm.

To confirm that the active form but not the full-length Sona are secreted *in vivo*, *Canton S* (*CS*) wing discs were stained to visualize extracellular Sona with Sona-Pro and -C antibodies, as described previously (see Materials and Methods)^40^. Both antibodies recognized extracellular Sona in the presumptive region of wing blade in the same region as lip-shaped but not in the DV midline region on the basal side of the disc proper (Fig. 5a). To prove that the pattern of extracellular Sona is authentic but not due to non-specific binding of Sona antibodies to ECM, Viking-GFP, Collagen IV protein fused to GFP commonly used as an ECM marker^41^, was also examined at the same confocal level. To distinguish intracellular Viking-GFP from extracellular Viking-GFP, the extracellular Viking-GFP was differentially stained with the extracellular staining protocol (Fig. 5b). At a glance, extracellular Viking-GFP was more evenly distributed than extracellular Sona-Pro, which was confirmed by the image at higher magnification (Supplementary Fig. S9).

A cross-section view of a wing disc also showed that extracellular Sona is present in the basal ECM of both disc proper and peripodial epithelium (Fig. 5c,d). However, there were clear differences between the structures recognized by the two Sona antibodies. The Sona-C antibody recognized some particulate structures that were not recognizable by the Sona-Pro antibody (Fig. 5e). These particulate structures may have active Sona that is devoid of the prodomain. In contrast, Sona-Pro antibody recognized a bunch of string-like structures (Fig. 5e”). Similar pattern was also observed in *nub* > *sona-HA* wing discs visualized with both Sona-Pro and HA antibodies (Fig. 5f). A magnified image revealed that a Sona form recognized by only anti-HA antibody, probably an active Sona, was localized in ECM as a separate identity (Fig. 5g).

### Loss of *sona* decreases the level of extracellular Wg

**Figure 6:**
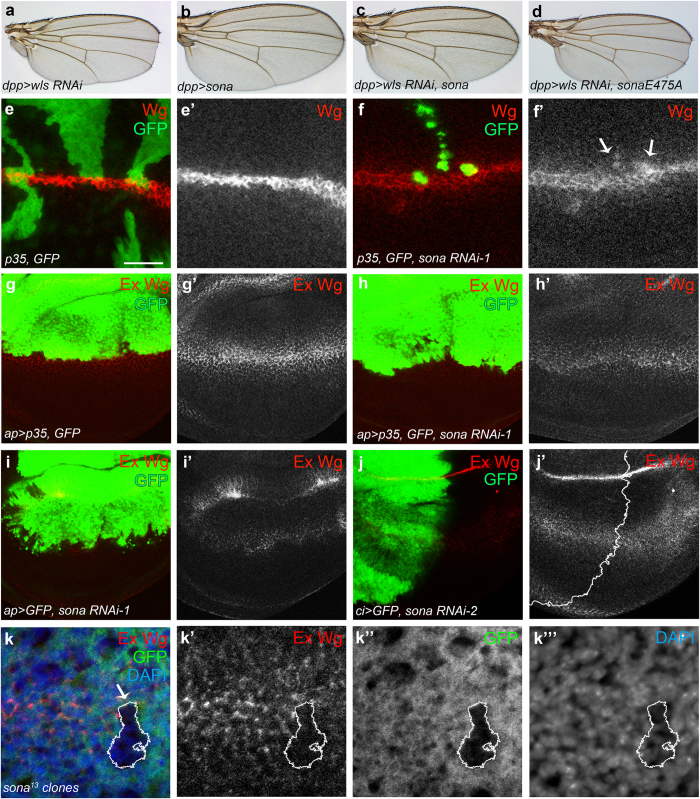
Loss of Sona increases the level of intracellular but decreases extracellular Wg. (**a**–**d**) Notched wing phenotype of *dpp* > *wls RNAi* flies (**a**) was rescued by expression of *sona* (**c**) but not *sonaE475* protease mutant form (**d**). *dpp* > *sona* wings had no phenotype (**b**). (**e,f**) Intracellular Wg in *sona RNAi* clones. *hs-Flp, P*[*Actin* > *yellow* > *Gal4; w* + ]/*+; UAS-p35, UAS-GFP*/+ wing disc as a control (**e**). *sona RNAi-1* clones in *hs-Flp, P*[*Actin* > *yellow* > *Gal4; w* +]/*+; UAS-p35, UAS-GFP/+; UAS-sona RNAi-1*/+ (**f**). Arrows indicate the *sona RNAi-1* clones with the increased level of intracellular Wg (f′). (**g**-**i**) Changes in the level of extracellular Wg by *sona RNAi* expression. *UAS-p35, UAS-GFP*/*apterous* (*ap*)*-Gal4;* +/*TM6 Tb* as a control (**g**), *UAS-p35, UAS-GFP*/*ap-Gal4; UAS-sona RNAi*/+ (**h**), and *UAS-GFP*/*ap-Gal4; UAS-sona RNAi*/+ (**i**). (**j**) Decrease in the extracellular level of Wg in the anterior region of *ci* > *sona RNAi-2* discs. The control is shown in Supplementary Fig. S11. (**k**) Decrease in the level of extracellular Wg in *sona*^*13*^ clones near the DV boundary. Scale bar: (**e**,**f**) 40 μm; (**g**–**j**) 60 μm; (**k**) 9.5 μm.

To understand the *in vivo* role of *sona*, we carried out a genetic screen with ethyl methanesulfonate (EMS) as a mutagen to obtain suppressors that could overcome the lethality by Sona overexpression. One of suppressors turned out to have a mutation in the *wls* gene. This study will be addressed in detail elsewhere (J.-H. W. and K.-O. C., manuscript in preparation). Wls is a transmembrane protein that is required for the secretion of Wnt/Wg^42^,^43^,^44^ and interacts with retromer complex for cycling from Golgi to the plasma membrane^45^,^46^,^47^,^48^,^49^. Consistent with the positive genetic interaction between *sona* and *wls*, *dpp* > *wls RNAi* flies had notched wing phenotype (n > 50 each), but *dpp* > *wls RNAi sona* flies had no notching (n = 20) at 18 °C (Fig. 6a–d). *dpp* > *wls RNAi sonaE475A* flies had notched wings, demonstrating that protease activity of Sona is essential to suppress the *wls RNAi* phenotype. The penetrance was 100% in all cases.

The result above prompted us to examine whether secretion of Wg is compromised in the clones expressing *sona RNAi-1.* Because loss of *sona* causes cell death (O. T. and K.-O. C., manuscript in preparation), the flp-out clones that coexpressed *sona RNAi* and caspase inhibitor *p35* were generated^50^. The level of intracellular Wg was increased in some clones compared to that of control clones (Fig. 6e,f). Not all clones showed the same phenotype, which suggests that only certain cells express Sona and need the function of Sona. This idea is consistent with the mosaic expression pattern of *sona* > *GFP* in wing pouch (Fig. 4h). When *sona RNAi-1* was expressed by the *apterous* (*ap*)*-Gal4* driver in the dorsal wing pouch, the level of intracellular Wg was also increased in the dorsal region regardless of coexpression with p35 (Supplementary Fig. S10). On the contrary, the level of extracellular Wg was overall decreased in *ap* > *sona* wing discs regardless of coexpression with p35 (Fig. 6g–i). When the *sona RNAi-2* was expressed by *cubitus interruptus* (*ci*)*-Gal4* in the anterior region of the wing disc, the extracellular level of Wg was lower in the anterior region than the posterior region (Fig. 6j; Supplementary Fig. S11). We also generated *sona*^*13*^ clones by the FLP-FRT method, and some of them exhibited the decreased level of extracellular Wg (Fig. 6k). Taken together, loss of *sona* increased the level of intracellular Wg but decreased that of extracellular Wg.

### Sona and Wg colocalize in Golgi and endosomal vesicles

**Figure 7:**
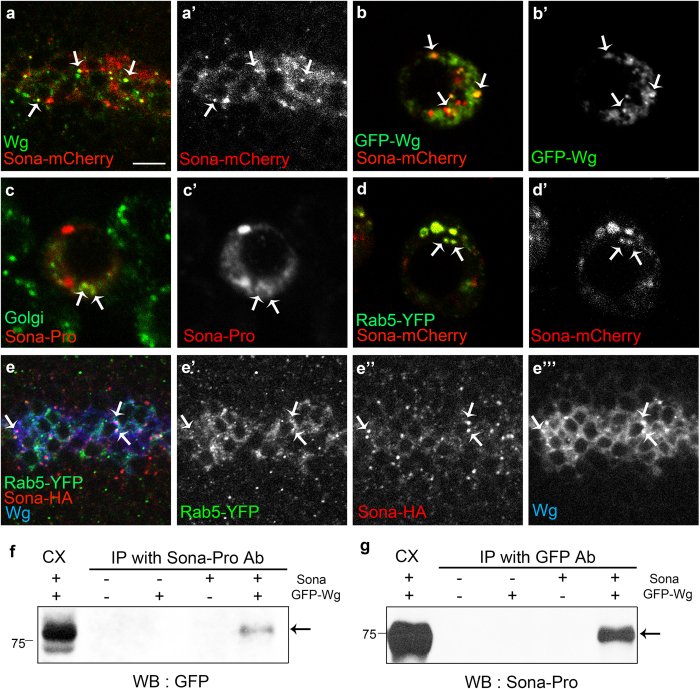
Sona and Wg colocalize in Golgi and endosomal vesicles. (**a**,**b**) Expression patterns of Sona-mCherry driven by *wg-Gal4* and endogenous Wg in the DV midline of wing discs (**a**) and those of GFP-Wg and Sona-mCherry in S2 cells transfected with *GFP-wg* and *sona-mCherry* cDNAs (**b**). Arrows point to a few representative vesicles containing both Wg and Sona-mCherry. (**c**) S2 cells transfected with *sona* cDNA were visualized for Sona (red) and P120 (green), a Golgi marker. Some vesicles containing both Sona and P120 are marked with arrows. (**d**) In S2 cells transfected with *rab5-YFP* and *sona-mCherry* cDNAs, Rab5-YFP^+^ Sona-mCherry^+^ vesicles were frequently present in the cytoplasm (arrows). (**e**) Vesicles containing Rab5-YFP and Sona-HA expressed by *wg-Gal4* in addition to endogenous Wg are marked with arrows. (**f,g**) Co-immunoprecipitation (IP) with cell extracts obtained from *GFP-wg* and *sona* transfected S2 cells. Cell extracts were IPed with Sona-Pro antibody, and GFP-Wg was detected with GFP antibody (arrow in **f**). Cell extracts were IPed with GFP antibody, and Sona was detected with Sona-Pro antibody (**g**). Scale bars: (**a**,**e**) 7.5 μm; (**b–d**) 5 μm.

Because both Sona and Wg are secreted proteins, and loss of *sona* decreased the level of extracellular Wg, we examined whether intracellular Sona and Wg are colocalized in S2 cells and wing discs. Both Wg and Sona-mCherry were enriched in the apical region of the *wg* > *sona-mCherry* wing disc (Supplementary Fig. S12), and about 60% (46/75, n = 4) of Sona-mCherry^+^ vesicles contained Wg and vice versa (Fig. 7a). Vesicles containing both Wg and Sona were also observed in S2 cells cotransfected with *GFP-wg* and *sona-mCherry* cDNAs (Fig. 7b). To address the nature of the vesicles containing both Wg and Sona, we checked whether Sona^+^ vesicles corresponded to Golgi vesicles in both wing discs and S2 cells. We found that some Sona^+^ vesicles are visualized with a Golgi marker P120^51^,^52^ in S2 cells (Fig. 7c).

Vesicles containing both Rab5-YFP and Sona-mCherry were frequently observed inside of S2 cells (Fig. 7d). About 30% (16/53) of Sona^+^ vesicles in the DV boundary region also contained Rab5-YFP in *wg* > *rab5-YFP* wing discs (Fig. 7e). Furthermore, Rab5-YFP^+^ Sona^+^ vesicles also contained Wg (Fig. 7e”’). Rab5, a small GTPase that regulates endocytic vesicle formation and early endosome fusion, is known to significantly co-localize with Wg and is involved in activation of Wg signaling^53^. Co-immunoprecipitation analysis showed that Sona and Wg are present in the same protein complex (Fig. 7f,g). Taken together, Sona and Wg may be secreted together in the same secretory pathway.

### Sona positively regulates Wg signaling

**Figure 8:**
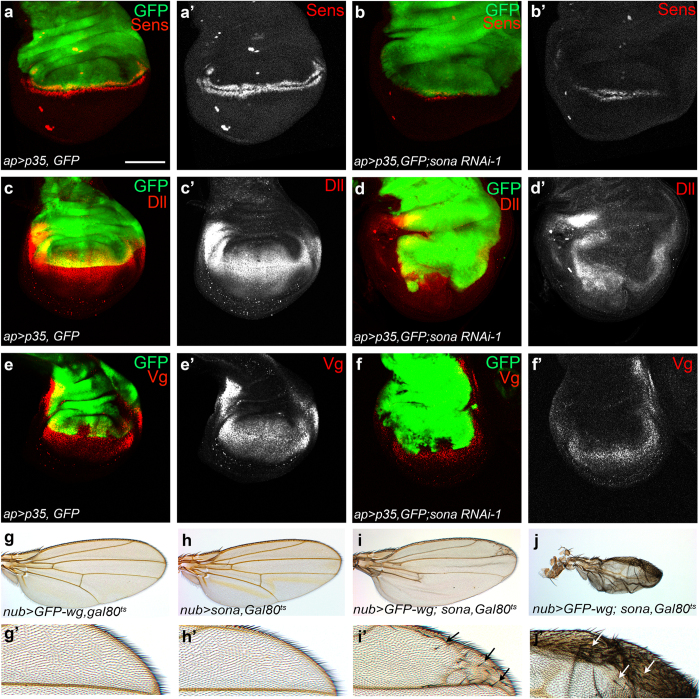
Sona enhances Wg signaling. (**a**–**f**) *UAS-p35, UAS-GFP*/*CyO-GFP; UAS-sona RNAi*/*TM6 Tb* and *ap-Gal4*/*CyO-GFP* flies were crossed and the larval progeny *UAS-p35 UAS-GFP*/*ap-Gal4*; +/*TM6 Tb* and *UAS-p35, UAS-GFP*/*ap-Gal4; UAS-sona RNAi*/+ were used as control and experimental animals, respectively. Patterns of Sens, Dll and Vg in wing discs. Anterior is left, and dorsal is up. Images of control discs (**a**,**c**,**e**) and experimental discs (**b**,**d**,**f**) are shown. Black and white images of Sens, Dll or Vg in (**a’**–**f’**). The expression region of ap-Gal4 is marked by GFP (green). (**g–j**) Wing phenotypes of flies transiently expressing GFP-Wg (**g**), Sona (**h**) or both (**i,j**) by *nub-Gal4* driver. The anterior margins of wings in (**g–j**) are magnified in (**g’**–**j’**). Ectopic bristles are marked with arrows (**i’**,**j’**). Genotypes of discs and adult wings are indicated at lower left. Scale bar: (**a**–**f**) 100 μm.

To further test whether Sona is required for Wg signaling, we checked the effect of *sona RNAi-1* on the expression of Sens, Dll and Vg in wing discs. P35 was coexpressed to prevent cell death by *sona RNAi* expression. Expression level of Sens was reduced in 80% (n = 9), and those of Dll (n = 46) and Vg (n = 25) were reduced in 100% of discs expressing *sona RNAi-1* (Fig. 8b,d,f), compared to the control discs (n > 10 for each) (Fig. 8a,c,e). Same results were obtained with *sona RNAi-2* driven by *ci-Gal4* (Supplementary Fig. S13). The expression level of Wg-LacZ was not decreased upon *sona RNAi* expression (Supplementary Fig. S14). Hence, Sona positively regulates Wg signaling by post-transcriptional regulation.

We then examined adult wings whether Sona can enhance Wg signaling. Since prolonged overexpression of either Sona or Wg caused lethality and developmental defects, we used Gal80^ts^ system to transiently express GFP-Wg and Sona^54^. Transient expression of either *GFP-wg* or *sona* with *nub-Gal4* during the late third larval and early pupal stage for 18 hours had no effect on the number of anterior bristles in adult wings or on lethality (n > 40 each; Fig. 8g,h). However, coexpression of *GFP-wg* and *sona* in the same culture condition caused lethality in about 85% of animals, and the survivors had multiple ectopic bristles near the anterior wing margin (n > 50; Fig. 8i,j). These phenotypes demonstrated that Sona promotes Wg signaling.

## Discussion

ADAMTSs are secreted metalloproteases that are known to be involved in mainly ECM remodeling. Among six ADAMTSs in the fly, Papilin is essential for the formation of basement membrane and fly development^32^, Stall functions in ovarian follicle formation and exhibits positive genetic interaction with Delta^35^, and ADAMTS-A is important for cell migration, especially in detaching cells from the apical ECM in salivary gland^34^. In this report, we have shown that Sona is a fly ADAMTS essential for fly development and survival. Transient coexpression of Sona and Wg increased the number of wing margin bristles, indicating that Sona is positively involved in Wg signaling. Accordingly, loss of *sona* decreased the level of Wg effector proteins as well as the level of extracellular Wg. Based on these results, we propose that Sona, as an ADAMTS, modifies yet unidentified protein(s) essential for Wg signaling.

During fly development, *sona* was transcribed at a high level in discrete regions in imaginal discs, which corresponded to the malformed regions in adult appendages of *sona* escapers (Figs 2 and 4). For instance, dorsal eye disc, the center of antenna disc, and outer ring of leg disc expressed the high level of *sona* transcripts, and *sona* escapers accordingly had disoriented ommatidial bristles in the dorsal eye, malformed arista, and kinked femur (Figs 2a–f and 4a–f). Wing disc also exhibited the complicated mosaic pattern of *sona* transcription, and adult wings of *sona* escapers were small and abnormally shaped (Figs 2g–j and 4g–i). Involvement of Sona in modulating the level of extracellular Wg may explain why these malformed adult structures are generated in *sona* escapers because Wg is specifically expressed in eye, wing and leg discs and determines the fate of organs^55^,^56^,^57^,^58^.

The genetic link between Sona and Wg signaling was identified in a genetic screen in which a *wls* allele could rescue the lethal phenotype caused by the overexpression of Sona. Likewise, wing notching by the loss of *wls* was rescued by overexpression of *sona* (Fig. 6c). Furthermore, the loss of *sona* decreased the level of extracellular Wg (Fig. 6g–i). Taken together, these results raised a possibility that Sona may be involved in Wg signaling by affecting Wg secretion. How may Sona positively regulate Wg secretion? To act on Wg secretion, Sona has to be activated intracellularly, and function in secretory pathways. It has been shown that the prodomains of most ADAMTSs are cleaved in trans-Golgi network to become active. Thus, activated intracellular Sona may cleave unidentified proteins involved in Wg secretion and thereby promote the secretion of Wg. Indeed, intracellular Sona was enriched in the apical region while extracellular Sona are more enriched in the basolateral region. Similarly, intracellular and extracellular Wg are enriched in the apical and basolateral regions, respectively^40^ (Fig. 5d; Supplementary Fig. S12). It has been recently shown that Wg is secreted to the apical side and then reentered cells by endocytosis, and then moves to the basal side and secreted by transcytosis^59^. It will be interesting to figure out whether Sona and Wg may be secreted together by transcytosis.

Besides the function of intracellular Sona for Wg secretion, presence of active Sona in conditioned medium of S2 cell culture suggests that extracellular active Sona may be involved in Wg signaling by modifying unknown ECM components (Fig. 3e). Immunocytochemical analysis of Sona confirmed that the active form of Sona devoid of the prodomain is present in basal ECM of wing discs (Fig. 5e–g). Therefore, active Sona may cleave ECM proteins that affect stability or activity of Wg. Well-studied ECM proteins essential for Wg signaling and formation of Wg gradient are Heparan sulfate proteoglycans (HSPG) such as Division abnormally delayed (Dally) and Dally-like (Dlp)^60^,^61^,^62^,^63^,^64^,^65^,^66^,^67^. These HSPGs can be modified by proteins such as Notum and Matrix metalloprotease 2 (Mmp2). Notum blocks Wg activity as α/β-hydrolase by modifying Dally and Dlp^68^, and Mmp2 cleaves Dlp to inhibit the interaction between Dlp and Wg^69^. Thus, Sona may act on these HSPGs or related ECM proteins to affect the stability or activity of extracellular Wg.

Extracellular Sona was highly localized in the presumptive region of wing blade in the basal ECM near the Collagen-IV containing region, but was present at lower level in the DV midline region where Wg is synthesized (Fig. 5). This data suggests that secreted extracellular Sona may not be diffused freely but restricted to a defined region by interacting with some ECM components. Another component of Wg signaling, Frizzled2 (Fz2), is also localized in the presumptive region of wing blade, but Frizzled3 (Fz3) is expressed exclusively in the DV midline^64^,^70^,^71^,^72^. Interestingly, Fz2 promotes but Fz3 attenuates Wg signaling^71^,^73^. Thus, these Fz proteins are strategically localized to bind the extracellular Wg in order to regionally regulate strength of Wg signaling. Similarly, Dally promotes but Dlp decreases Wg signaling in the DV midline^63^,^64^, and extracellular Dlp is present at a lower level in the DV midline region^60^. Taken together, specific localization of these Wg signaling components in ECM may be essential for modulating Wg signaling with regional specificity in wing discs.

Involvement of Sona in Wg signaling raises a possibility that some mammalian ADAMTSs may also be involved in Wnt signaling. Some mammalian ADAMTSs are known to function as positive factors for tumor invasion and progression^74^,^75^,^76^. Overexpression of Wnts or downstream components of Wnt signaling also induces various tumors such as colon cancer, breast cancer, and leukemia^77^,^78^. Wnt signaling is also essential for the growth and remodeling of bones and connective tissues^79^,^80^,^81^,^82^. Overlapping functions of ADAMTSs and Wnt signaling supports our view that some mammalian ADAMTSs may be linked to Wnt signaling. Further work on identifying the intracellular or extracellular substrate(s) of Sona is required to fully understand how Sona is positively involved in Wg signaling.

## Materials and Methods

### *Drosophila* strains, transgenic lines and generation of ectopic clones

We carried out imprecise excision of a P element, P{GawB}CG9850[NP5284], and obtained 9 *sona* deletion mutants by screening about 200 flies. Deficiency lines (*Df*(*2R*)*X32, Df*(*2R*)*23, Df*(*2R*)*3-659*) that uncover the *sona* gene were used to confirm *sona* deletions. We generated two *sona RNAi* lines targeting different regions of the *sona* coding region (Fig. 1b; Supplementary Fig. S1) and several *UAS* transgenic flies: *UAS-sona*, *UAS-sona-HA*, *UAS-sonaE475A*, and *UAS-sona-mCherry. UAS-GFP-wg*^83^, *wg-Gal4*^64^ and *viking-GFP *41 were kindly provided. All other lines were obtained from Bloomington stock center.

For generation of ectopic clones, *hs-Flp*, *UAS-sona-mCherry*/*TM6 Tb* males and *y w ; P*[*Actin* > *CD2* > *Gal4; w* + ]/*Cyo-GFP ; UAS-GFP*/*TM6 Tb* females were crossed, their progeny were raised at 18 °C until 48 hr AEL, and then heat shocked at 37 °C for 1 hour as described^3^. They were then kept at 18 °C until dissection.

### DNA constructs

*sona* cDNA was originally obtained from a two-hybrid cDNA library screening^84^ using the first and second PDZ domains of Discs-Large (Dlg) as a bait^85^,^86^. Out-of-frame fusion between the *Gal4* activating domain and the portion of *sona* gene that encodes the carboxyl terminus fortuitously generated a perfect but gratuitous PDZ binding motif, which let Sona to be identified in the screen.

To generate *sona-HA* construct, *HA* tag was attached at Gly638 by removing stop codon with *HpaI* digestion and ligating amplified *HA* tag. *pUAST-sona-HA-mCherry* was generated by tagging *mCherry* at the downstream of *HA* tag in the *pUAST-sona-HA* plasmid. *pUAST-sonaE475A* was generated by changing GAA (glu) to GCA (ala) by site-directed mutagenesis. *pAc-GFP-wg* was constructed by recombining the *pAc5.1* vector and *GFP-wg* that was derived from *MK33-GFP-wg* (a gift from J.P. Vincent, unpublished).

### Northern blot and *in situ* hybridization

For Northern analysis, RNA was prepared as described and 20 μg of RNA each was loaded in 2.2 M formaldehyde containing agarose gel^87^. The RNAs were transferred to nitrocellulose and probed with ^32^P-labeled 1.9 kb *XhoI* fragment that covers the 3′ half of the *sona* cDNA for Northern analysis. The same 1.9 kb *XhoI* fragment was linearlized and amplified by polymerase chain reaction using one primer to make digoxigenin-labelled single-stranded DNA probe for *in situ* mRNA hybridization as described^88^.

### Cell culture and transfection

*Drosophila* S2 cells were grown in M3 media (Sigma-Aldrich) supplemented with 10% IMS (Sigma-Aldrich) at 25 °C. Transfections were carried out with transfection reagents effectene (Qiagen) or cellfectin (Invitrogen) according to the manufacturers’ instructions. For each transfection, a total of 1–2 μg DNA was used.

To establish *S2 sona-HA* cell line that stably expresses *sona-HA*, Hygromycin B selection system was used according to the manufacturers’ instructions (Invitrogen life technologies). Briefly, 4 × 10^6^ *S2* cells were cotransfected with total 1 μg of two plasmids, pAC sona-HA and pCoHygro (19:1 ratio), for 3 days. Then, the culture medium was changed to selective medium containing 150 μg/ml of hygromycin B (Invitrogen). The selective medium was replaced every 5 days, and the *sona-HA* cell lines were established after 3 weeks. The established *sona-HA* cell line was maintained in selective medium containing Hygromycin B.

### Antibodies and immunocytochemistry

We generated Sona-Pro antisera in rabbits using GST fusion protein with 245 amino acid residues representing 20th to 264th, and Sona-C antisera in mice using 378 amino acid residues representing 262th to 639th of Sona protein as antigens. Both Sona-Pro and Sona-C antisera were purified by affinity purification using MBP- Pro and GST-C, respectively. Following dilutions were used for Western analysis: Sona-Pro, 1:5,000; HA (Santa Cruz, rabbit), 1: 250, and for immunocytochemistry: Sona-Pro, 1:300~500; Sona-C, 1:300~500; Golgi (Calbiochem, mouse), 1:200; GFP (Abd Serotec, sheep), 1:100; Senseless (gift from H. Bellen, guinea pig), 1:1,000; HA (Roche, rat), 1:150; HA (Santa Cruz, rabbit), 1:100; Dlg (rabbit), 1:500; Dlg (DSHB, mouse), 1:100; Wg (DSHB, mouse), 1:100; Dll (Santa Cruz, goat), 1:100; Vg, 1:100 (gift from S. Carroll, rabbit).

Fly larvae were cultured at 25 °C unless stated otherwise. For intracellular staining, imaginal discs were dissected and stained as described^85^. The samples were incubated with primary antibodies in washing buffer (50 mM Tris pH6.8, 150 mM NaCl, 0.5% NP-40, 1 mg/ml BSA) for overnight at 4 °C or room temperature and washed with washing buffer several times. Then, samples were incubated with secondary antibodies and washed several times before mounting. For extracellular staining of Wg and Sona, we followed the method as described^87^. The wing imaginal discs were dissected in M3 media at 4 °C. The samples were incubated with about 3–4 fold more primary antibodies than for intracellular staining in cold M3 media for 2 hrs. Then, samples were washed with cold M3 and PBS twice, fixed for 40 minutes in 4% paraformaldehyde/PBS, and then socked in non-detergent blocking buffer (60 mM Tris pH6.8, 150 mM NaCl, 5 mg/ml BSA) for 3 hrs at 4 °C. Samples were then incubated with secondary antibodies with non-detergent washing buffer (50 mM Tris pH6.8, 150 mM NaCl, 1 mg/ml BSA) and washed with non-detergent blocking buffer four times and detergent blocking buffer twice before mounting. For HA and GFP extracellular staining, the following antibodies, HA (Roche, rat, 1:30) and GFP (Abcam, rabbit, 1:30) were used. Fluorescent images were captured using Zeiss LSM laser scanning confocal microscope and presented using Adobe Photoshop.

### Western analysis and co-immunoprecipitation

For Western analysis, samples were mixed with 4 × SDS sample buffer and boiled at 95 °C for 10 min. Samples were then separated by 10–12% SDS-PAGE and transferred to the nitrocellulose membrane (Millipore). Membranes were blocked with 5% nonfat milk in TBST buffer (10 mM Tris pH 7.4, 0.8% NaCl, 0.1% Tween-20), and probed with the antibody. After washing membranes with TBST several times, membranes were incubated with horseradish peroxidase (HRP)-conjugated secondary antibody in TBST with 5% nonfat milk. After washing, protein bands were visualized using the ECL system (AbFrontier).

For co-immunoprecipitation, cells were lysed in HEPES buffer (20 mM HEPES, 70 mM KCl, 2 mM DTT, 0.1% NP40, 8% Glycerol, 1 mM PMSF, 10 mM EDTA, 10 mM EGTA, and protease inhibitor cocktail (Roche)) on ice. The lysates were precleared by incubating with protein G-sepharose beads (Amersham Bioscience) for 30 min at 4 °C. A new set of G-sepharose beads were incubated with anti-GFP (Abcam, rabbit) or Sona-Pro (rabbit) for coupling at room temperature for 2 hrs. The precleared lysates were then incubated with coupled protein G-sepharose beads for overnight at 4 °C. The protein G-sepharose beads were washed with HEPES buffer and Western blots were performed as described.

## Additional Information

**How to cite this article**: Kim, G.-W. *et al.* Sol narae (Sona) is a *Drosophila* ADAMTS involved in Wg signaling. *Sci. Rep.* **6**, 31863; doi: 10.1038/srep31863 (2016).

## Supplementary Material

Supplementary Information
