# Supplementary material for: Sol narae (Sona) is a Drosophila ADAMTS involved in Wg signaling
Source: Sci Rep. 2016 Aug 18;6:31863. doi: 10.1038/srep31863 (PMC4989167; doi:10.1038/srep31863)

Sol narae (Sona) is a *Drosophila* ADAMTS involved in Wg signaling

Go-Woon Kim†§, Jong-Hoon Won†, Ok-Kyung Lee, Sang-Soo Lee, Jeong-Hoon Han, Orkhon Tsogtbaatar, Sujin Nam, Yeon Kim, and Kyung-Ok Cho*

Supplementary Information

Table 1. Loss and gain of function phenotypes of *sona*.

UAS- sona, *UAS-sona RNAi-1* and *UAS-sona RNAi-1* were crossed with various *Gal4* lines and the phenotypes of their progeny are summarized.

Table 2. Lethality of sona mutants rescued by overexpression of Sona-PA.

To obtain the total number of progeny including two parental types and both rescued and unrescued nonparental types, both empty pupal cases and dead pupae were counted. The dead pupae should have nonparental genotype. *CyO/CyO* flies are embryonic lethal, so are not counted in total progeny count.

The ratio between paternal, maternal and nonparental genotype as adults should be approximately 1:1:1 if the % rescue is 100%. Under incomplete rescue, a certain fraction of the nonparental progeny dies. Because homozygous *sona* mutants also die during larval stage, not all nonparental types can be counted in total progeny count. Therefore, the number of nonparental types is underestimated, and, accordingly, the % rescue in this table is somewhat overestimated.

Figure S1. Sequence comparison of ADAMTSs and generation of *sona* mutant lines.

The sequence alignment of human ADAMTS-16, mouse ADAMTS-16, *C. elegans* MIG-17 and *Drosophila* Sona. The color bars indicate different domains of Sona as shown in Figure 1**a**. The light blue rectangle indicates the conserved active site of the protease domain in

ADAMTS family. The black thick arrow indicates the regions for two *RNAi* lines of *sona*. The vertical arrow indicates glutamic acid (E) residue that was changed to alanine (A) in SonaE475A mutation.

Figure S2. Interommatidial bristles are disoriented especially in dorsal eye.

(**a**) Dorsal eye of *w*^{1118} as a control. (**b**) Dorsal eye of *sona*^{47}/ *sona*^{18} transheterozygous adult. Arrow indicates the disoriented dorsal interommatidial bristles. These phenotypes were also observed in of *sona*^{47}/*sona*^{13} and *sona*^{13} /*sona*^{18} transheterozygous escapers.

Figure S3. SonaE475A does not have any effect on wing development.

The adult wings of *nub-Gal4/+* flies (**a**), *nub>sona* flies (**b**) and *nub>sonaE475A* flies (**c**). The flies were cultured at 25°C (**a**,**c**) or 18°C (**b**).

Figure S4. Sona is absent in the larval extract of *sona* mutants.

Full-length Sona (arrow) is absent in homozygous *sona*^{13}, *sona*^{18} and *sona*^{47} larval extracts. The bands marked with asterisks are nonspecific, and their appearance differs among batches of purified antibodies

Figure S5. Sona is expressed in photoreceptor clusters.

(**a**,**b**) The eye disc of *sona>GFP* was stained with Sona-Pro and GFP antibodies. The squared region in **a** was magnified in **b**, and black-and-white images of GFP and Sona-Pro are shown in **b'** and **b''**, respectively. (**c**,**d**) Similar structure in wild-type eye disc is visualized with Sona-C antibody in **c. d** is DAB staining for Sona-C antibody as described[1]. (**e**-**h**) Negligible level of signasl was obtained with Sona-Pro and Sona-C antibodies in *sona*^{18} homozygote mutant wing disc (**f**, **h**). Scale bar: **a**, **c**, 30 μm; **e**-**h**, 60 μm.

Figure S6. Sona-Pro and Sona-C antibodies recognized Sona.

(**a-d**) The wing discs of *ptc>GFP, sona-HA* were used to detect HA-tagged Sona with HA antibody, carboxyl region of Sona with Sona-C antibody, and prodomain region of Sona with Sona-Pro antibody. Both Pro and C antibodies recognized spots in the *ptc* region that were also recognized by HA antibody (**b',b",d',d"**). **b** or **d** was magnified image of the square on **a** or **c**. Scale bar: **a**,**c**, 100 μm.

Figure S7. Overexpression of Sona-HA and Sona-mCherry by nub-Gal4 generated malformed wings

The adult wing phenotypes of *UAS-sona-HA* (**a**) and *UAS-sona-mCherry* (**b**) expressed by *nub-Gal4.* Genotypes are indicated at lower.

Figure S8. mCherry tag present in Sona-mCherry is not cleaved in both CX and CM.

Full-length Sona-HA and Sona-mCherry are present in CX. The bands marked with asterisks are nonspecific. In CM, active forms of Sona-HA (black arrow) and Sona-mCherry (red arrow) but no other small cleaved fragments are present. The protein bands were visualized with anti-HA antibody.

Figure S9. The localization pattern of extracellular Sona and Vkg-GFP

Sona and Vkg-GFP are expressed in the wing pouch. Vkg-GFP is more widely distributed than Sona. Scale bar: **a-c,** 40 μm.

Figure S10. The level of intracellular Wg is increased by *sona RNAi* expression.

(**a-c**) A wing disc expressing *p35* and *GFP* by *ap-Gal4* as a control (**a**), *GFP* and *sona RNAi* (**b**), and *p35, GFP* and *sona RNAi* (**c**). Scale bar: **a-c,** 60 μm.

Figure S11. The extracellular level of Wg in control *ci>GFP* disc.

Control disc for Figure 6j. No change in the extracellular level of Wg in *ci>GFP* disc. Scale bar: **a-c,** 60 μm.

Figure S12. Intracellular Wg and Sona-mCherry in *wg>sona-mCherry* discs are enriched in the apical region.

Cross-section view of the sample used to obtain Fig.7a. Scale bar: 9.5 µm.

Figure S13. The expression levels of both Dll and Vg are reduced in the anterior region of *ci> sona RNAi-2* discs.

sona RNAi-2 can reduce the level of Dll and Vg, similar to *sona RNAi-1*. Scale bar: **a-b,** 60 μm.

Figure S14. Loss of Sona does not affect the transcriptional level of *wg*.

sona RNAi and *GFP* were expressed by *en-Gal4* in *wg-lacZ* transgenic flies and the wing discs were stained for LacZ and GFP. Scale bar: 60 μm.

Reference

1 Choi, K. W. & Benzer, S. Rotation of photoreceptor clusters in the developing Drosophila eye requires the nemo gene. *Cell* **78**, 125-136 (1994).

Supplementary Table and Figures

Table 1

Gal4 driver	Phenotype of Sona over-expression	Phenotype of *sona* RNAi-1 expression	Phenotype of *sona* RNAi-2 expression
engrailed-Gal4	Not hatching	Pupal lethal (>95%)	Pupal lethal (>60%)<br>weak adults<br>minor defects such as folding in posterior wing
patched-Gal4	Not hatching	Pupal lethal (>95%)<br>Loss of ACV,<br>Distance between L3 and L4 shortened	Pupal lethal (>60%)<br>weak adults
nubbin-Gal4	No wing,<br>Pupal lethal	Small wing, loss of vein, D/V detachment	Small wing
GMR-Gal4	No eye,<br>Pupal lethal	Disordered Eye bristle	N/D
wg-Gal4	Small wing,<br>disrupted ocelli bristles	Notched wing, No eye, shorten legs or missing legs	N/D
30A-Gal4	Pupal lethal	N/D	N/D
sona-Gal4	Laval to pupal lethal(>90%),<br>impaired larval trachea, small and weak adults	Pupal lethal (>90%),<br>Twisted legs,<br>Disordered eye bristle, small salivary gland, weak adult	N/D
hh-Gal4	N/D	Early larval lethal	Late pupal lethal (>90%)
act-Gal4	N/D	Early larval lethal	Early pupal lethal

Table 2

**paternal genotype**	**maternal genotype**	**genotype of rescued progeny**	**% rescue**
nub-Gal4 sona^{47} */CyO-GFP*	*sona*^{47} *UAS-sona /CyO*	*nub-Gal4 sona*^{47}*/ sona*^{47} *UAS-sona*	35.7% (12 rescued adults in total 101 progeny)
ptc-Gal4 sona^{47} */CyO-GFP*	*sona*^{47} *UAS-sona /CyO*	*ptc-Gal4 sona*^{47} */ sona*^{47} *UAS-sona*	23.0% (8 rescued adults in total 104 progeny)
sona^{47} */CyO-GFP*	*sona*^{47} *UAS-sona /CyO*	*sona*^{47} */sona*^{47} *UAS-sona*	0% (0 rescued adult in total 128 progeny)

Figure S1

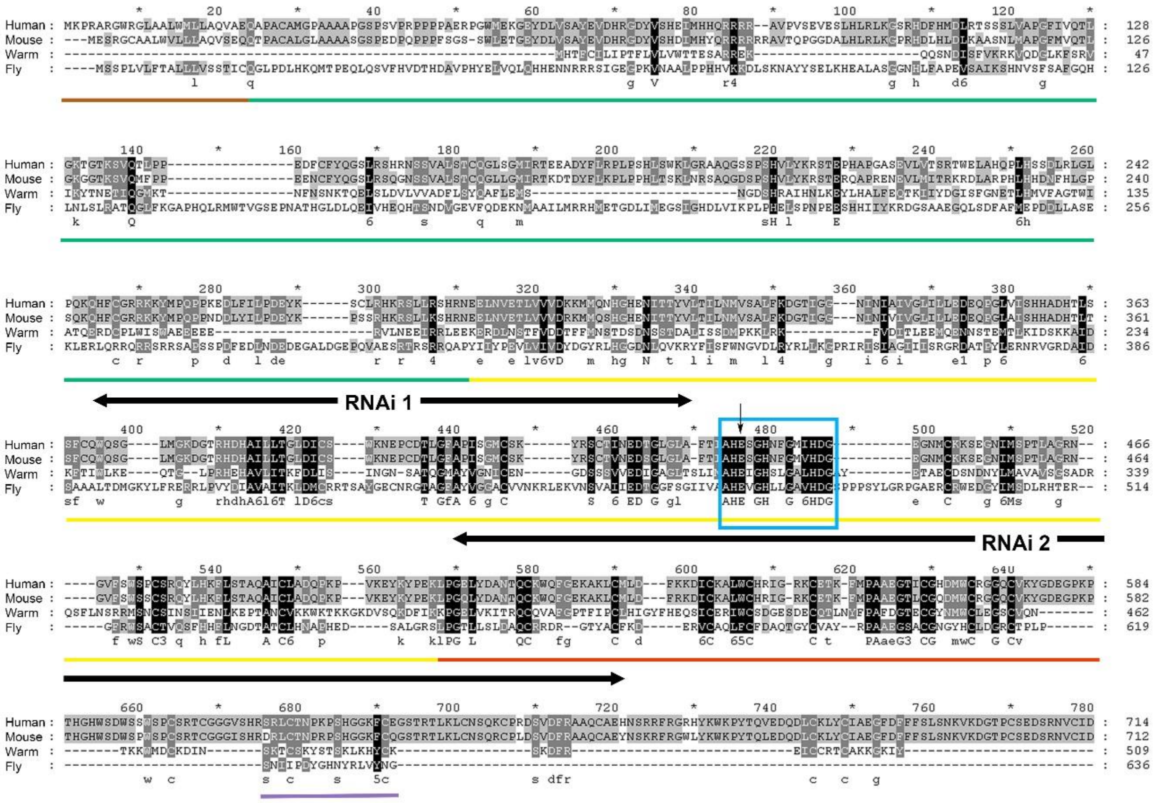

Figure S2

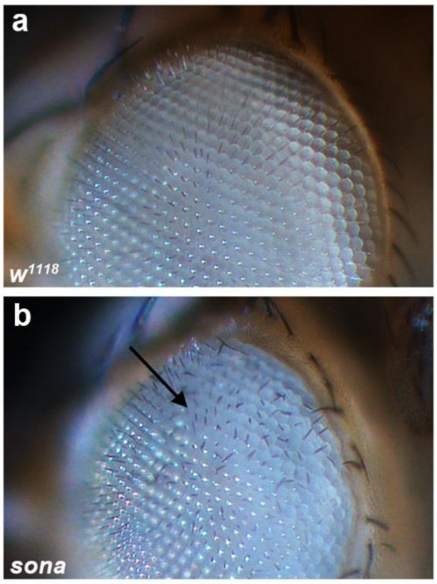

Figure S3

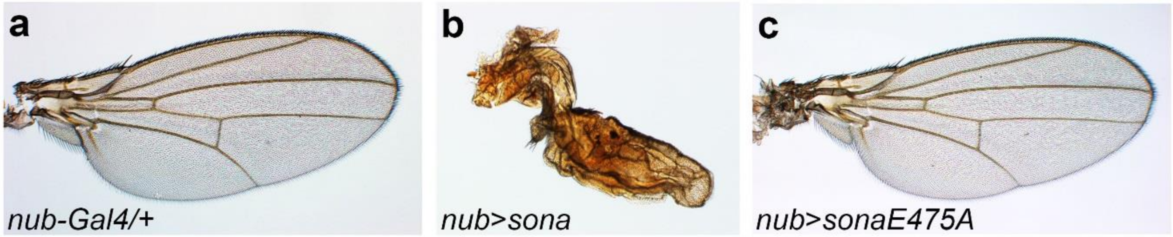

Figure S4

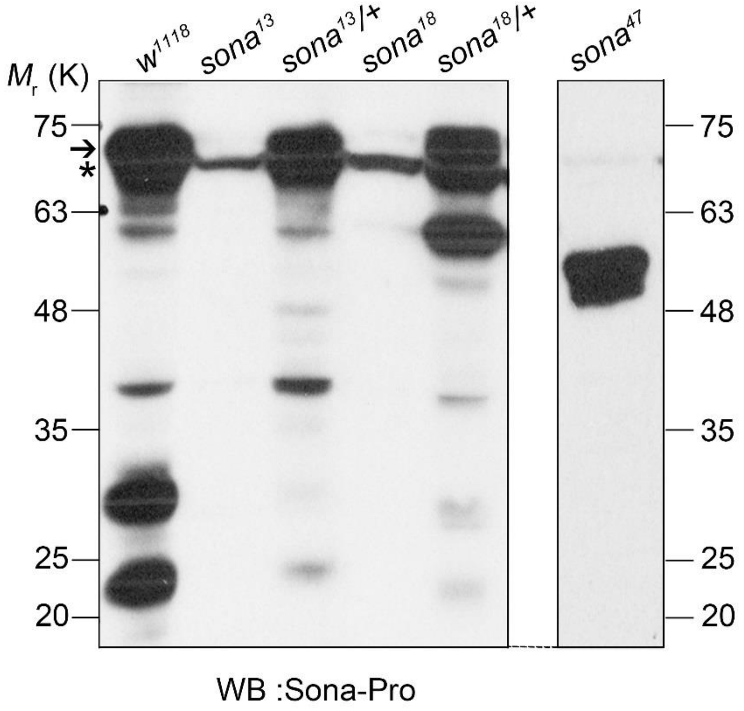

w^{1118}
$sona^{13}$
$sona^{13}/+$
$sona^{18}$
$sona^{18}/+$
$sona^{47}$
M_r (K)
75
63
48
35
25
20
75
63
48
35
25
20
*
WB :Sona-Pro

Figure S5

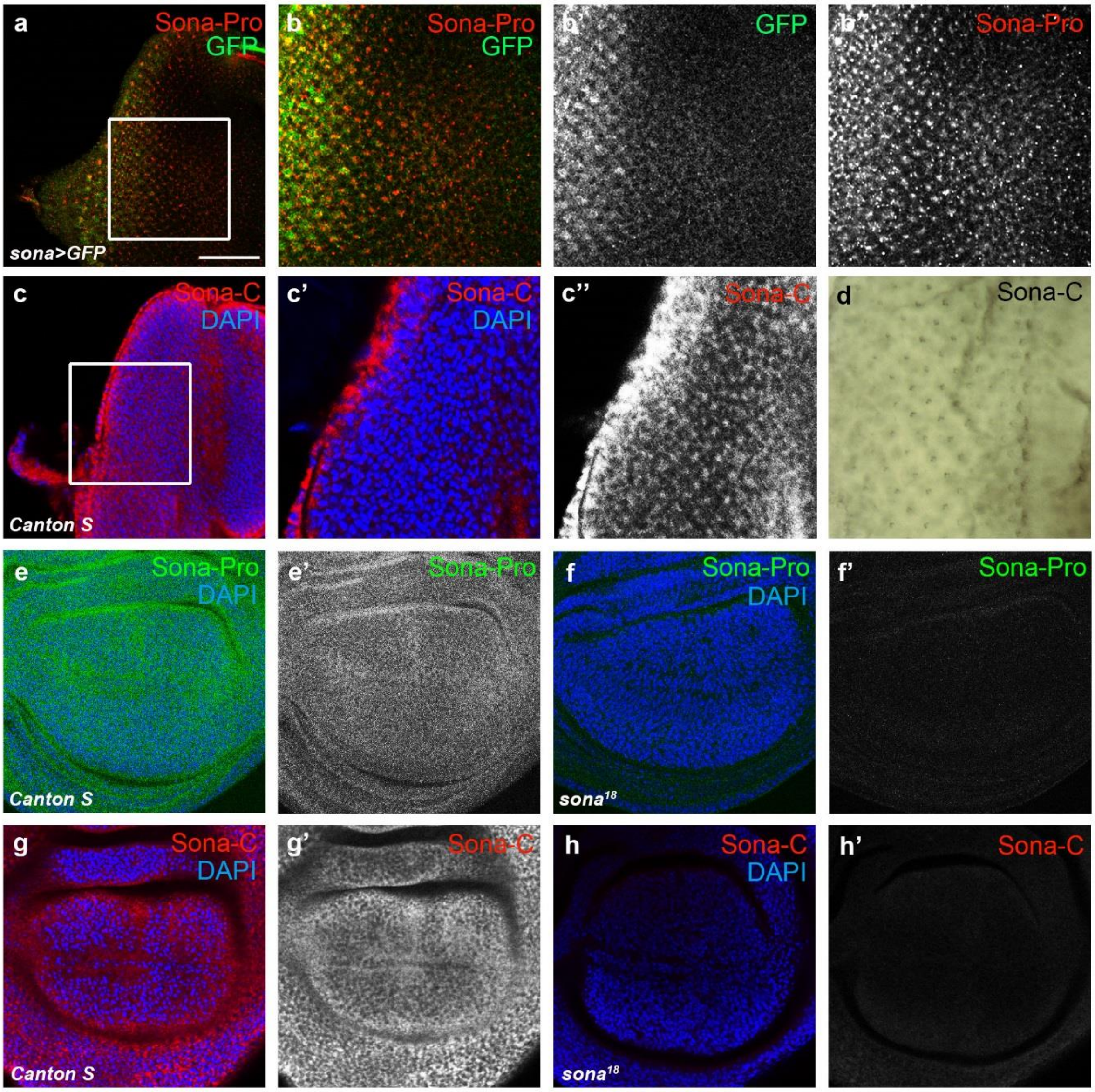
a
Sona-Pro
GFP
sona>GFP
b
Sona-Pro
GFP
b'
GFP
b''
Sona-Pro
c
Sona-C
DAPI
Canton S
c'
Sona-C
DAPI
c''
Sona-C
d
Sona-C
e
Sona-Pro
DAPI
Canton S
e'
Sona-Pro
f
Sona-Pro
DAPI
sona18
f'
Sona-Pro
g
Sona-C
DAPI
Canton S
g'
Sona-C
h
Sona-C
DAPI
sona18
h'
Sona-C

Figure S6

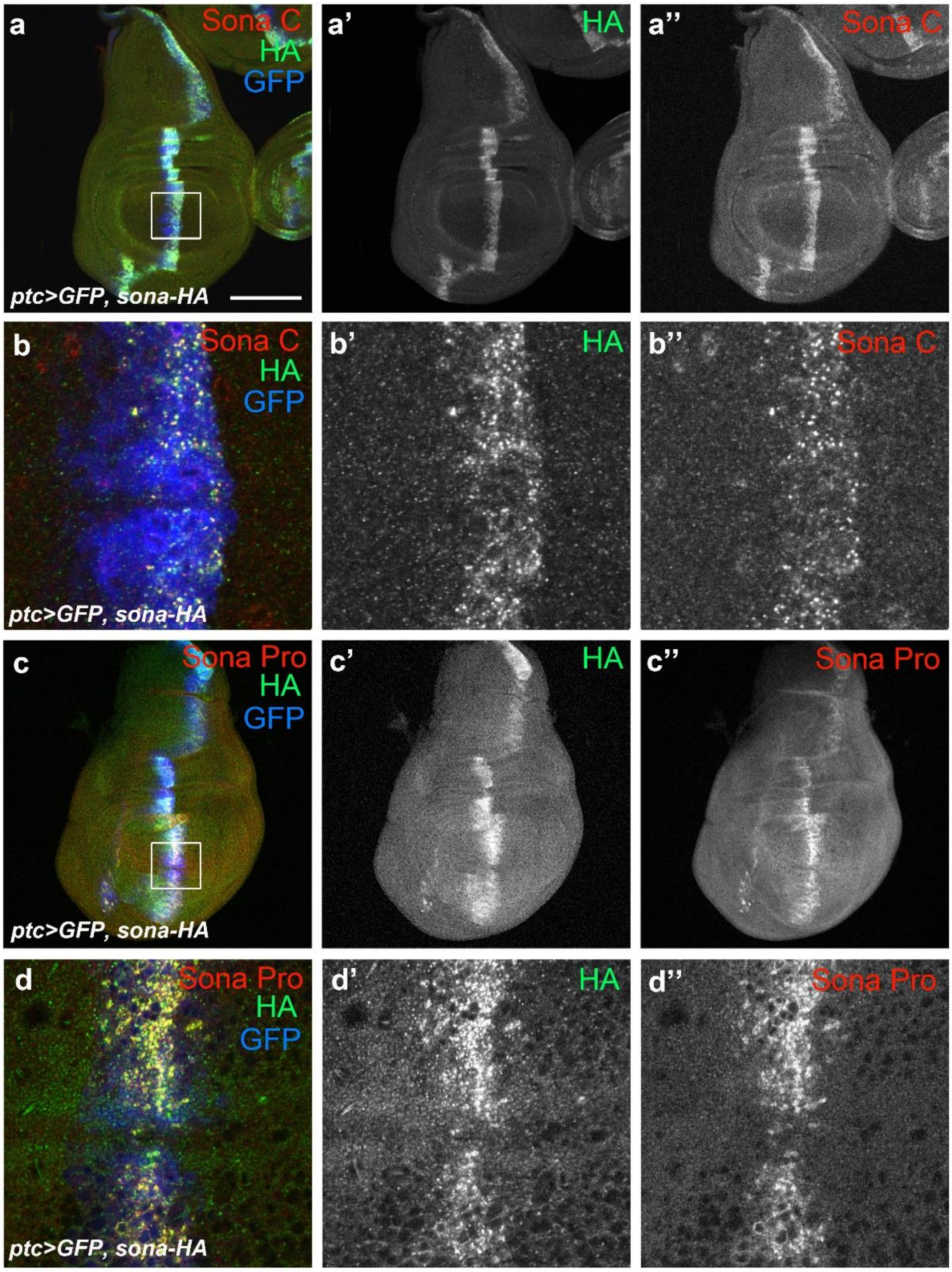
a
Sona C
HA
GFP
ptc>GFP, sona-HA
a'
HA
a''
Sona C
b
Sona C
HA
GFP
ptc>GFP, sona-HA
b'
HA
b''
Sona C
c
Sona Pro
HA
GFP
ptc>GFP, sona-HA
c'
HA
c''
Sona Pro
d
Sona Pro
HA
GFP
ptc>GFP, sona-HA
d'
HA
d''
Sona Pro

Figure S7

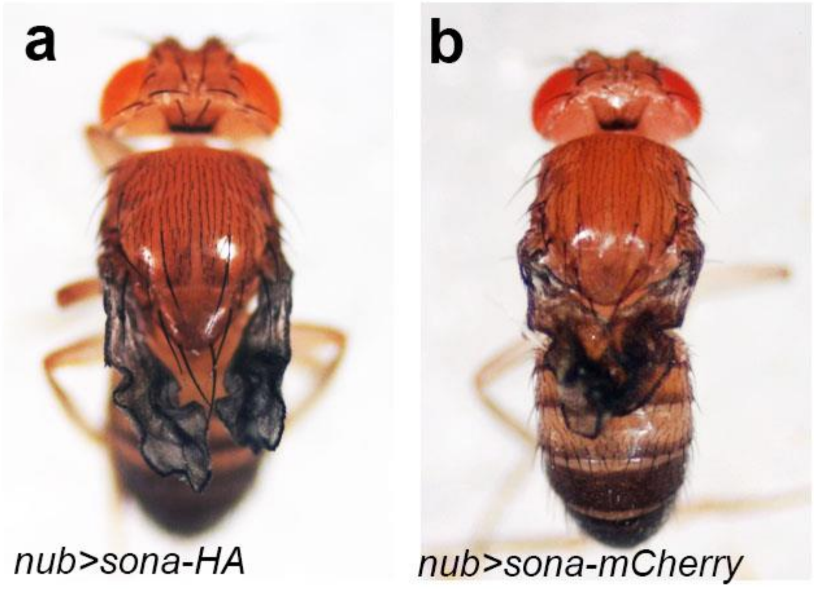

Figure S8

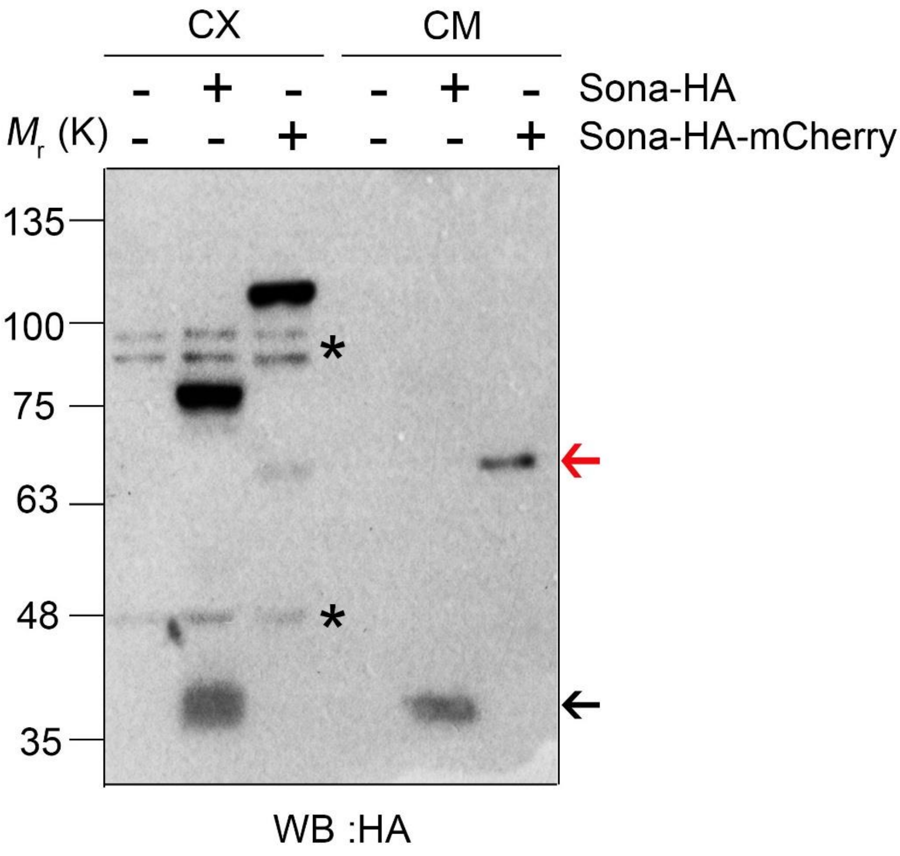

Figure S9

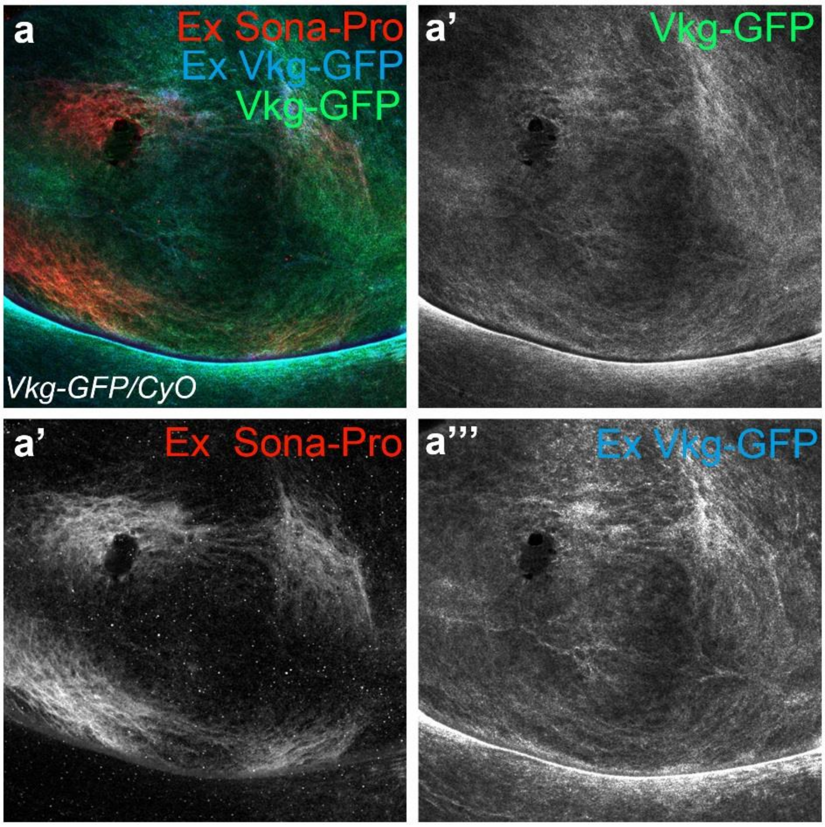
a
Ex Sona-Pro
Ex Vkg-GFP
Vkg-GFP
Vkg-GFP/CyO
a'
Vkg-GFP
a'
Ex Sona-Pro
a'''
Ex Vkg-GFP

Figure S10

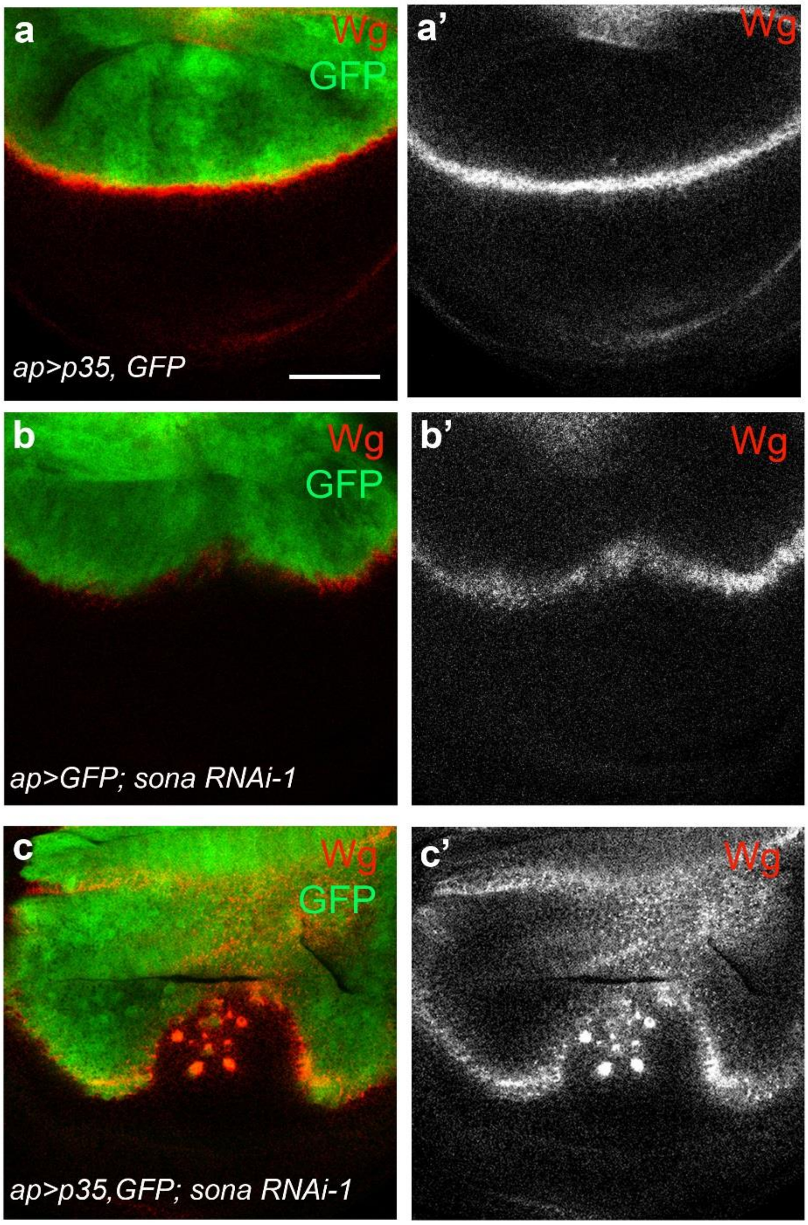
a
Wg
GFP
ap>p35, GFP
a'
Wg
b
Wg
GFP
ap>GFP; sona RNAi-1
b'
Wg
c
Wg
GFP
ap>p35,GFP; sona RNAi-1
c'
Wg

Figure S11

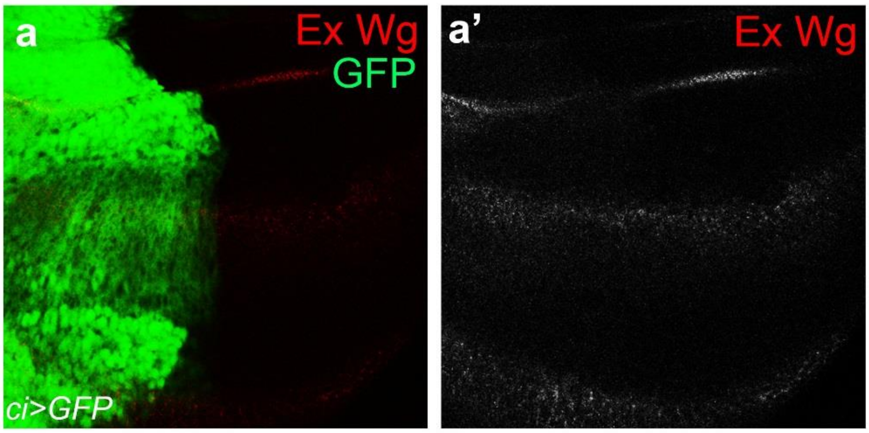

Figure S12

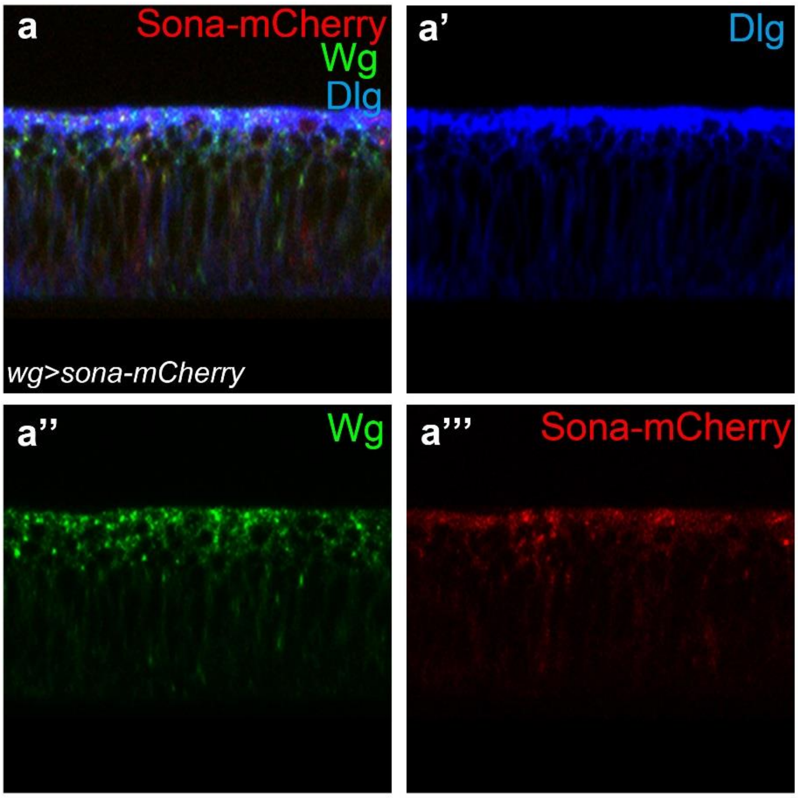

Figure S13

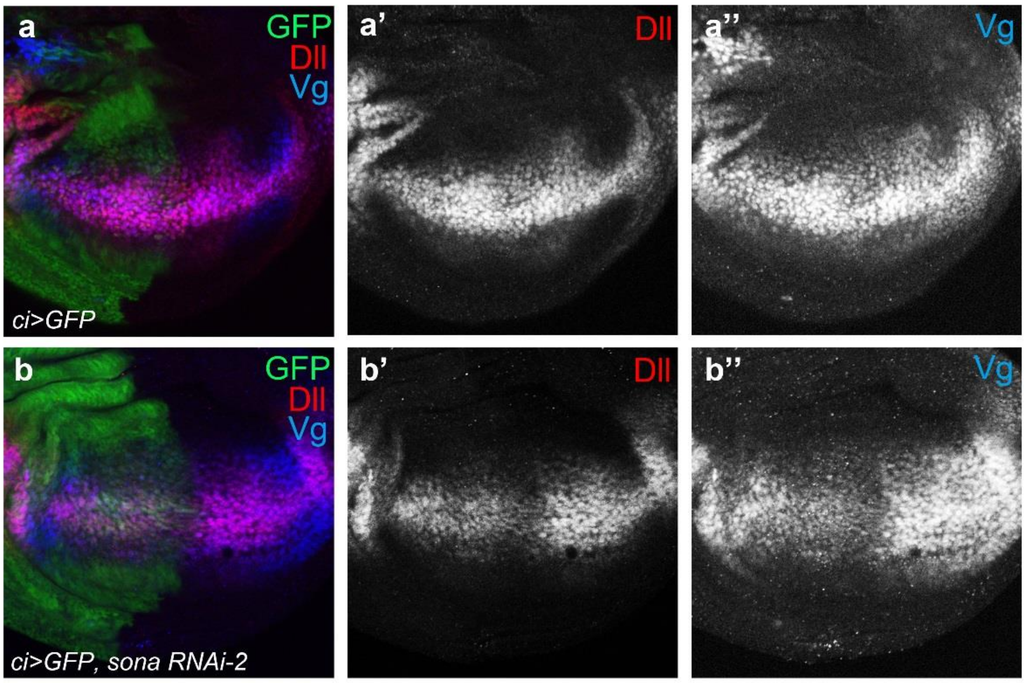
a
GFP
Dll
Vg
ci>GFP
a'
Dll
a''
Vg
b
GFP
Dll
Vg
ci>GFP, sona RNAi-2
b'
Dll
b''
Vg

Figure S14

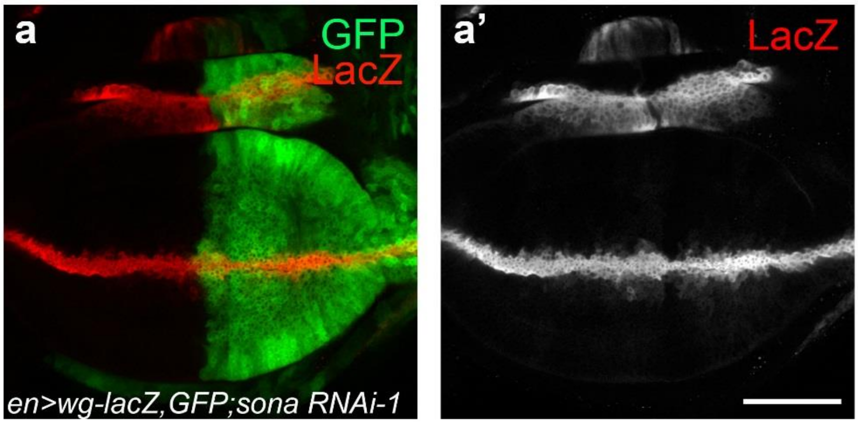

Supplement: Supplementary Information [file srep31863-s1.pdf]
